# Host Transcriptome Analysis of *Spodoptera frugiperda* Larvae Parasitized by *Microplitis manilae*

**DOI:** 10.3390/insects14020100

**Published:** 2023-01-17

**Authors:** Ahamaijiang Gulinuer, Binglin Xing, Lei Yang

**Affiliations:** 1 Sanya Nanfan Research Institute, Hainan University, Sanya 572024, China; 2 School of Tropical Crops, Hainan University, Sanya 572024, China

**Keywords:** parasitoid, *Microplitis manilae*, *Spodoptera frugiperda*, metabolism, immunity

## Abstract

**Simple Summary:**

The interactions between parasitoids and their host have always attracted much attention, and parasitoids manipulate the host physiology to benefit the survival and development of their offspring. It is critical to analyze these physiological regulatory mechanisms. In this study, using RNA-sequencing analysis, we clarified the effects of parasitization on host gene expression of *Microplitis manilae*, a dominant larval parasitoid of *Spodoptera frugiperda*. Several differentially expressed genes were identified in *S. frugiperda* larvae at 2 h, 24 h, and 48 h post-parasitization. Among these, many genes were implicated in host metabolism and immunity. Further qPCR results involving 20 differentially expressed metabolism, and immune-related genes were consistent with the expression profiles of the RNA-seq data, which confirmed the physiological manipulation of *M. manilae* on host *S. frugiperda*. These results contribute to defining the molecular basis of wasps’ successful parasitization and facilitate their application.

**Abstract:**

It has been extensively found that parasitoids manipulate host physiology to benefit the survival and development of their offspring. However, the underlying regulatory mechanisms have not received much attention. To reveal the effects of parasitization of the larval solitary endoparasitoid *Microplitis manilae* (Hymenoptera: Braconidae) on host *Spodoptera frugiperda* (Lepidoptera: Noctuidae), one of the most destructive agricultural pests in China, deep-sequencing-based transcriptome analysis was conducted to compare the host gene expression levels after 2 h, 24 h, and 48 h parasitization. A total of 1861, 962, and 108 differentially expressed genes (DEGs) were obtained from the *S. frugiperda* larvae at 2 h, 24 h, and 48 h post-parasitization, respectively, compared with unparasitized controls. The changes in host gene expressions were most likely caused by the injection of wasp parasitic factors, including PDVs, that were injected along with the eggs during oviposition. Based on the functional annotations in GO and KEGG databases, we revealed that most DEGs were implicated in host metabolism and immunity. Further analysis of the common DEGs in three comparisons between the unparasitized and parasitized groups identified four genes, including one unknown and three prophenoloxidase (PPO) genes. Moreover, 46 and 7 common DEGs involved in host metabolism and immunity were identified at two or three time points after parasitization, respectively. Among these, most DEGs showed increased expressions at 2 h post-wasp parasitization while exhibiting significantly decreased expression levels at 24 h post-parasitization, demonstrating the expression regulations of *M. manilae* parasitization on host metabolism and immune-related genes. Further qPCR verification in 20 randomly selected DEGs confirmed the accuracy and reproducibility of the gene expression profiles generated from RNA-seq. This study reveals the molecular regulatory network about how host insects respond to wasp parasitism, laying a solid foundation for revealing the physiological manipulation of wasp parasitization on host insects, which facilitates the development of biological control practices for parasitoids.

## 1. Introduction

*Spodoptera frugiperda* (J. E. Smith), commonly known as fall armyworm (FAW), belongs to the *Spodoptera* (Lepidoptera: Noctuidae), which originated from the tropical regions of the western hemisphere from the United States to Argentina [1]. Adult FAWs have solid migratory ability. Since their migration to Yunnan province in early 2019, the FAW colonized a wide range of corn fields in southern China. and rapidly spread to 26 provinces in approximately one year [2]. FAW has the characteristics of high fertility and adaptability, which possess a wide range of hosts. In addition, FAW larvae are highly polyphagous and have host ranges of more than 353 plants, including cereal crops and economic crops, such as corn, sorghum, rice, sugarcane, and tobacco [3]. Over the past few years, chemical insecticides have been the primary way to control this pest. However, the long-term application of pesticides has led to high levels of resistance, environmental pollution, and pesticide residue.

Biological control is a method that uses beneficial organisms in nature, such as parasitic wasps, predatory natural enemies, and pathogenic microorganisms, to dampen the oscillations of agricultural pests [4]. Evidence has suggested that parasitic wasps are among the most important biological control agents for controlling pests and have been extensively used [5]. Our previous research has established that more than 170 species are parasitizing FAW, showing their great potential to manage FAW [6]. Among these, braconids accounted for more than 35%, constituting the most species-rich group.

As unique parasitic organisms among hymenopteran insects, parasitoids are classified into endoparasitoids and ectoparasitoids according to their greatly varied oviposition patterns [7]. Endoparasitoids lay eggs into the hemocoel of host insects, while ectoparasitoids oviposited on the surface of host insects [8]. Both endoparasitoids and ectoparasitoids introduce various maternal factors into the host insects while laying eggs. Of these factors, venoms, polydnaviruses (PDVs), virus-like particles (VLPs), and ovarian secretion are mainly included [9,10,11,12], and they cooperate to counteract the host resistances, ultimately ensuring the offspring’s survival. It has been well documented that the physiological processes of host insects, mainly involving immunity, development, and metabolism, were substantially regulated by parasitization [9,13,14,15].

The rapid development of omics technology provides great convenience for revealing the physiological interactions between parasitoid wasps and host insects during their co-evolution. Of these, the transcriptome is currently one of the most frequently used methods to uncover the regulatory network underlying parasitization. To date, numerous studies have used high-throughput sequencing to examine parasitization’s effects on their hosts’ gene expression. For instance, the genome-wide gene expression of *Drosophila* larvae in response to wasp attack was first demonstrated by Wertheim et al. (2005) [16], showing that a series of candidate genes associated with innate immune responses exhibited remarkable altered expression. In addition, Schlenke et al. (2007) [17] performed a whole genome microarray analysis to show that the generalist and specialist wasp employed different infection strategies to defeat the immune response of host *Drosophila*. Subsequently, RNA-seq was conducted after a parasitoid attack among four *Drosophila* hosts, which found a core set of upregulated genes implicated in immune response [18]. In another major study, Danneels et al. (2013) [19] found that the responsive genes related to metabolism, development, and immune response of the host fly were significantly induced after parasitization by *Nasonia vitripennis* (Hymenoptera: Pteromalidae). In parallel, a key study analyzing the effect of parasitism on host gene regulation was that of Chevignon et al. (2015) [20], in which transcriptomes were obtained from the unparasitized and parasitized *Manduca sexta* (Lepidoptera: Sphingidae) and the specific effects of *Cotesia congregata* (Hymenoptera: Braconidae) parasitism on host gene expression were revealed. Recently, to gain insight into the effects of parasitization by *Aulacocentrum confusum* (Hymenoptera: Braconidae) on host *Glyphodes pyloalis* (Lepidoptera: Pyralidae), Sheng et al. (2021) [21] conducted a transcriptome analysis between parasitized and unparasitized host and identified a battery of differently expressed immune-related genes in parasitized larvae. In a follow-up study, RNA-seq was used to quantify the gene expression levels of the host midgut after parasitization, revealing that the metabolic pathway-related and immune genes were significantly upregulated [22].

*Microplitis manilae* (Ashmead) (Hymenoptera: Braconinae) is a preponderant solitary larval endoparasitoid parasitizing *Spodoptera* species with high parasitic efficiency [23]. The developmental period of *M. manilae* from egg to larvae ranged between 24–48 h at 25 °C [24]. Upon 6–8 d post-parasitization, the mature larvae of *M. manilae* drill out of the host, and wasp cocoons form along with the death of host insects. In contrast, the unparasitized *Spodoptera* host aged 6–8 d develops into the third instar larvae. Similar to many braconid wasps, *M. manilae* is equipped with multiple virulence factors, such as PDV and venom (Unpublished data), which are indispensable for successful parasitism [25]. Although there are many lines of research addressing the biological characteristics of this wasp, little has been explored into the physiological responses of host FAW post-parasitization by *M. manilae*. Accordingly, the main objective of this study is to investigate the transcriptional responses in *M. manilae*-parasitized FAW host larvae and identify the differentially expressed genes (DEGs) followed by qPCR verification. Our present study will contribute to a better understanding of the physiological interactions between the larval parasitoid and its host using the *M. manilae*–*S. frugiperda* system, thus facilitating the biological control practice for lepidopteran pests.

## 2. Materials and Methods

### 2.1. Insects

FAW and *M. manilae* were originally collected from the corn field of the Hainan province of China in 2019, and the laboratory colonies were respectively established [24]. Briefly, FAW larvae were fed on a standard artificial diet (Patent no. 201921652702.2). The adults were maintained on 10% hydromel (*v*/*v*) and were allowed to lay eggs on medical gauzes. The newly hatched host larvae were used for parasitization within 48 h (first instar). The wasps were raised under the controlled conditions of 25 ± 1 °C, 14 L: 10 D photoperiod, and 55 ± 5% relative humidity inside plastic bottles (10 cm × 5 cm). Once they emerged, we fed the adults with 10% hydromel (*v*/*v*).

### 2.2. Sample Collection

The emerged wasp adults within 24 h were collected and fully mated for 24 h followed by parasitizing the FAW first instar larvae for 2 h with a wasp/host ratio of 1:5. Subsequently, we removed the wasps and maintained the parasitized host larvae according to the above conditions. Fifteen parasitized FAW larvae at 2 h, 24 h, and 48 h post-parasitization were served as the experimental groups, and samples from 15 same-aged unparasitized host larvae were used as the control groups. Each group was repeatedly sampled thrice. All samples were collected into a centrifuge tube with 1 mL TRIzol Reagent (Invitrogen, Carlsbad, CA, USA) and fully ground in liquid nitrogen for subsequent RNA extraction.

### 2.3. RNA Extraction and Transcriptome Sequencing

We extracted the total RNA using TRIzol reagent (Invitrogen, Carlsbad, CA, USA) following the manufacturer’s instructions. Then, the RNA purity (OD 260/280) was checked using a Nanodrop 2000 spectrophotometer (NanoDrop products, Wilmington, DE, USA), followed by the detection of RNA degradation and DNA contamination with an Agilent Bioanalyzer 2100 (Agilent Technologies, Santa Clara, CA, USA). After the quality inspection, 1 µg RNA was used as the input to gather mRNA molecules by poly-T oligo-attached magnetic beads. We constructed transcriptome sequencing libraries using the Illumina^®^ RNA Library Prep Kit (NEB, Beijing, China) according to the manufacturer’s instructions. AMPure XP Beads (Beckman Coulter, Beverly, CA, USA) were used to purify the synthesized cDNA. All 18 library preparations were sequenced on the Illumina HiSeq2500 platform with the 150 bp paired-end sequencing protocol (Novogene Bioinformatics Technology Co., Ltd., Beijing, China). Raw data of FAW transcriptome were deposited in the NCBI SRA database (Project number: PRJNA818900).

### 2.4. Transcriptomic Data Analysis

Raw reads were processed to remove the reads containing adaptors, Poly-N, and low-quality reads. The filtered clean reads were mapped to the FAW reference genome (SRA project number: PRJNA647344) using Hisat2 tools [26], and the longest transcripts with a perfect match were chosen as the unigenes for further annotation. All unigenes were annotated on NCBI non-redundant (NR), SwissProt, and Clusters of orthologous groups for complete eukaryotic genome (KOG) databases using BlastX (e-value < 1 × 10^−5^). To annotate the potentially significant enriched metabolic pathways, we used KOBAS software to map the unigenes to the Kyoto Encyclopedia of Genes and Genomes (KEGG) database (e-value < 1 × 10^−10^) [27]. Subsequently, Gene Ontology (GO) enrichment analysis was performed using the GOseq R packages based on Wallenius non-central hyper-geometric distribution (e-value < 1 × 10^−6^) [28].

### 2.5. DEGs Identification

The fragments per kilobase of transcript per million mapped reads (FPKM) were calculated to obtain the expression levels of each unigene using the software Bowtie 2 and eXpress [29,30]. The differentially expressed unigenes between the parasitized and unparasitized host larvae at 2 h, 24 h, and 48 h were identified using the DESeq2 software with strict screening thresholds of corrected *p*-value < 0.05 and |log_2_ Fold change(FPKM)| > 1 [31]. We used TBtools software to plot the hierarchical heatmap of DEGs [32]. To identify the DEGs at two or three time points, the Venny 2.1 tool was used (https://bioinfogp.cnb.csic.es/tools/venny/index.html, accessed on 1 December 2022).

### 2.6. qPCR Analysis

Quantitative PCR was performed using the CFX96™ Real-Time PCR Detection System (Bio-Rad, Hercules, CA, USA) with the ChamQ SYBR qPCR Master Mix Kit (Vazyme, Nanjing, China). The specific qPCR primers were designed by AlleleID 6 software (PREMIER Biosoft, Palo Alto, CA, USA), and actin was used as the reference gene (Table S1). qPCR program was set as 3 min at 95 °C and 40 cycles for 15 s at 95 °C and 30 s at 60 °C. Each measurement was repeated thrice. Gene expression levels between the two groups were calculated based on the comparative 2^-ΔΔCt^ method [33]. The expression fold changes of DEGs were visualized using GraphPad Prism 7.0 software (GraphPad, San Diego, CA, USA), and data were analyzed by unpaired two-tailed Student’s *t*-Test. Pearson’s correlation method was used to evaluate the association between RNA-seq and qPCR. In brief, log_2_ Fold change(qPCR) values were plotted against the log_2_ Fold change(FPKM) values. All statistical significances were tested using Data Processing System (DPS) package version 9.50 and marked with different asterisks (*: *p* < 0.05, **: *p* < 0.01, ***: *p* < 0.001) [34].

## 3. Results

### 3.1. Analyses of Host Larvae Transcriptome after M. manilae Parasitization

**Figure 1:**
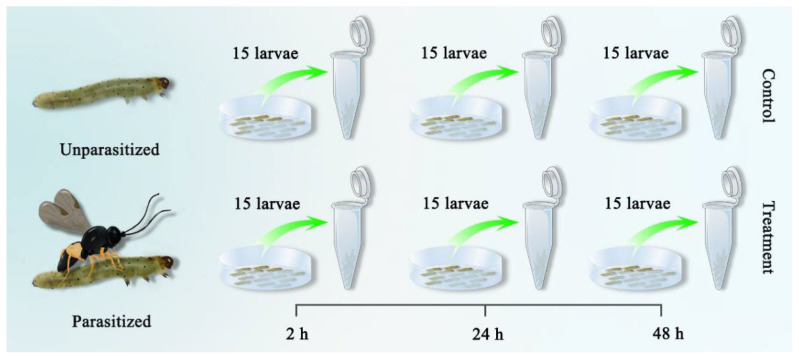
Experimental set-up of transcriptome sequencing. Transcriptome data were generated from the FAW larvae upon 2 h, 24 h, and 48 h parasitization, and the unparasitized larvae at the same developmental stages served as the controls. Three comparisons were performed, including 2 h post-parasitized host vs. 2 h unparasitized host (P2 vs. NP2), 24 h post-parasitized host vs. 24 h unparasitized host (P24 vs. NP24) and 48 h post-parasitized host vs. 48 h unparasitized host (P48 vs. NP48).

Six cDNA libraries of host larvae were separately generated and then sequenced, including the samples of 2 h, 24 h, and 48 h post-parasitization, and unparasitized FAW larvae at the same developmental stages served as the controls to comprehensively reveal the regulations of *M. manilae* parasitization on host *S. frugiperda* (Figure 1). Of these, three independent biological replicates were conducted at each time point. We obtained a range of 5.15–8.32 Gb sequenced raw reads per sample, which generated 113.78 Gb of clean data in total (Table S2). Quality inspection showed that the Q30 values of all samples were larger than 90.44 %, implying the high quality of transcriptome data. In the following, the clean data were mapped to the reference genome of FAW. The results indicated that at least 74.19% of reads had matches in the FAW genome. Finally, 32,749 assembled unigenes were obtained.

### 3.2. Identification of DEGs

**Figure 2:**
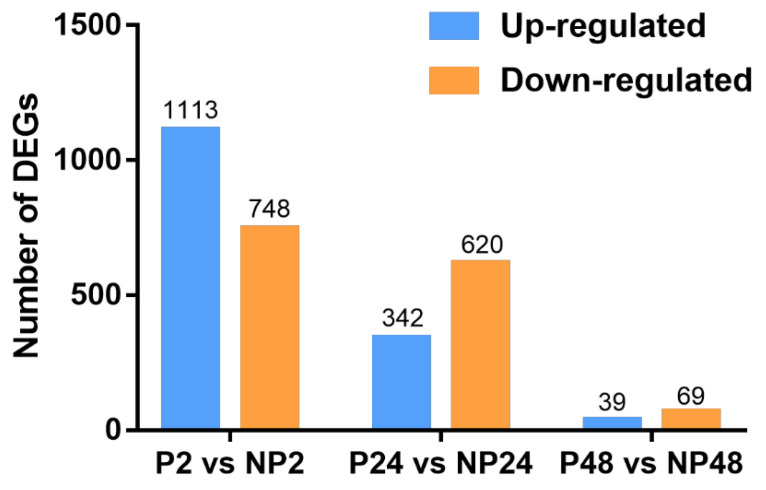
Identification of DEGs between the unparasitized and parasitized FAW larvae. Numbers of DEGs identified from the P2 vs. NP2, P24 vs. NP24, and P48 vs. NP48 groups. The upregulated and downregulated DEGs were presented in the blue and orange columns, respectively.

To define a robust set of DEGs in transcriptome data between the unparasitized and parasitized groups at each time point, we set the criteria as corrected *p*-value < 0.05 and |log_2_ Fold change(FPKM)| > 1. As a result, presented in Figure 2, 1861 DEGs, including 1113 upregulated and 748 downregulated items, were identified upon 2 h parasitization compared with the control (Table S3). In contrast, 342 and 620 unigenes were respectively upregulated and downregulated in the parasitized FAW at 24 h compared with the 24 h unparasitized host (Figure 2 and Table S4). Also, 108 unigenes were differentially expressed after 48 h parasitization, and of these, 39 upregulated and 69 downregulated items were included (Figure 2 and Table S5). Overall, these results suggested a significant decline in the number and proportion of DEGs with the time extension after *M. manilae* parasitization.

### 3.3. Functional Characterization of DEGs

**Figure 3:**
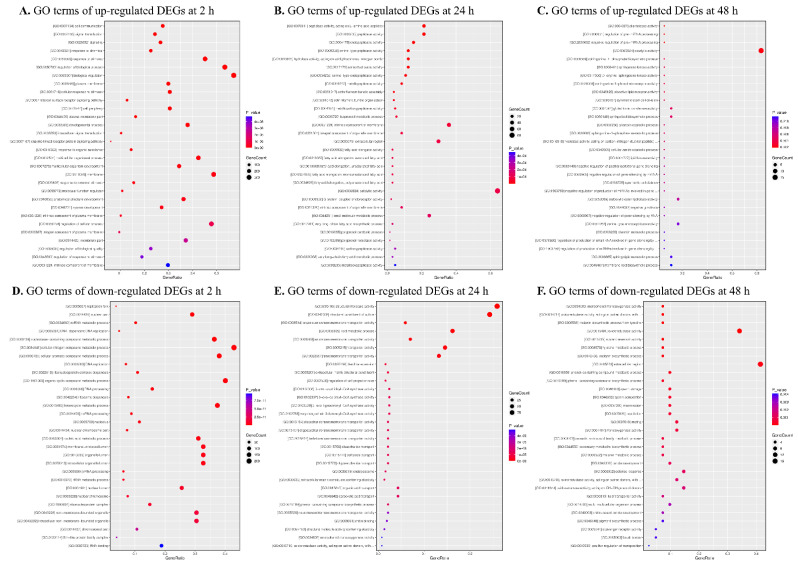
GO functional categories of DEGs obtained from the comparisons of P2 (**A**) vs. NP2 (**D**), P24 (**B**) vs. NP24 (**E**), and P48 (**C**) vs. NP48 (**F**) groups. The rich factor represents the ratio of DEGs in a certain GO category. The size and color of the dots, respectively, indicate the range of gene count and *p*-value mapped to the corresponding terms.

We sorted the 2931 DEGs into three categories: biological process, molecular function, and cell component, according to their annotations in the GO consortium database. In P2 vs. NP2 comparison, genes categorized as having “biological regulation” (GO: 0065007) and “cellular nitrogen compound metabolic process” (GO:0034641) were most abundant in the upregulated and downregulated terms, respectively (Figure 3A,D). Unlike the results of 2 h post-parasitization with *M. manilae*, the terms “catalytic activity” (GO: 0003824) and “structural molecule activity” (GO:0005198) represented the most upregulated and downregulated categories in P24 vs. NP24 comparison, independently (Figure 3B,E). Similarly, the dominant term of 39 upregulated DEGs identified from the P48 vs. NP48 group was also enriched for the GO category “catalytic activity” (GO: 0003824) (Figure 3C). In addition, the most downregulated DEGs were assigned to the “extracellular region” term (GO:0005576) (Figure 3F). From the obtained results, it was concluded that a large proportion of DEGs in the P2 vs. NP2 group were classified into different GO terms compared with the P24 vs. NP24 or P48 vs. NP48 groups, implying the occurrence of an obvious shift in the physiological processes of host FAW upon 24 h parasitization.

To better reveal the function of DEGs on the molecular interaction, reaction, and relation networks upon *M. manilae* parasitization, their participation in KEGG pathways was figured out (Table 1). What stood out in Table 1 was that the top 10 enriched KEGG pathways in DEGs after 2 h parasitization were assigned into the metabolism, genetic information processing, human diseases, and cellular processes categories. Similarly, it was found that the most enriched category of DEGs in the P24 vs. NP24 group also pertained to the metabolism pathway. In addition, the items with the most representation by the DEGs identified from the P48 vs. NP48 group were categorized into the metabolism, cellular processes, and human disease pathways. Based on the KEGG annotations, we revealed that most DEGs were implicated in the host metabolism, which provided a novel insight into the physiological regulation of host FAW larvae upon *M. manilae* parasitization.

### 3.4. Identification of Common DEGs after M. manilae Parasitization

**Figure 4:**
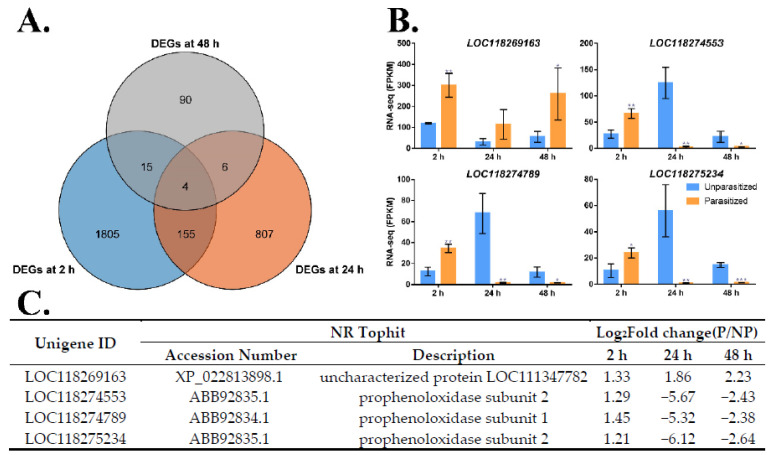
Identification of common DEGs at 2 h, 24 h, and 48 h post-parasitization with *M. manilae*. (**A**) Venn diagram of DEG numbers between unparasitized and parasitized FAW larvae at 2 h, 24 h, and 48 h. (**B**) The comparisons of FPKM in four common DEGs between unparasitized and parasitized host larvae and data were presented as mean ± SE (*n* = 3). (**C**) List of the four common DEGs and their functional annotations in the NR database.

The next set of questions aimed to identify the common DEGs in three groups. It was apparent that 1805, 807, and 90 DEGs were specifically presented in the FAW larvae at 2 h, 24 h, and 48 h post-parasitization with *M. manilae*, respectively (Figure 4A). Closer inspection of the results showed that the comparisons of “P2 vs. NP2 and P24 vs. NP24”, “P2 vs. NP2 and P48 vs. NP48,” and “P24 vs. NP24 and P48 vs. NP48” groups identified 159, 19, and 10 common DEGs, individually. In addition, the expression levels of four DEGs were simultaneously changed upon 2 h, 24 h, and 48 h parasitization. Based on their top hits in the NR database, the four genes were respectively annotated as the genes encoding one unknown protein (LOC118269163) and three prophenoloxidases (PPOs) (LOC118274553, LOC118274789, and LOC118275234) (Figure 4C). Among these, the unknown gene (LOC118269163) showed a consistently higher expression in the parasitized FAW larvae at three time points (Figure 4B). Moreover, a similar expression pattern was observed in three PPO genes, which were remarkably upregulated in the FAW larvae after 2 h parasitization, while there were significant downregulation at 24 h and 48 h post-parasitization. Together all results, it was suggested that *M. manilae* parasitization regulated the gene expression of FAW larvae associated with immunity.

### 3.5. Common Metabolism and Immune-Related DEGs

**Figure 5:**
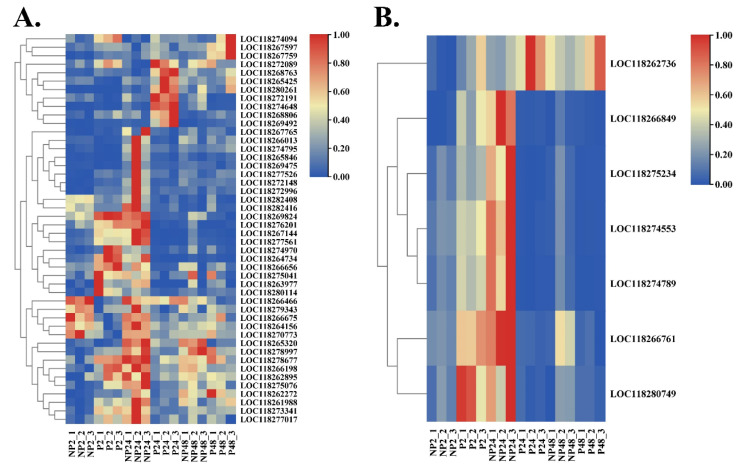
Expression profiles of DEGs involved in host metabolism and immunity. (**A**) Expression profiles of metabolism-related genes in host FAW upon *M. manilae* parasitization. (**B**) Expression profiles of immune-related genes in host FAW upon *M. manilae* parasitization. The higher and lower expression levels are respectively shown in red and blue colors based on the log_2_(FPKM) values.

The next section of the survey was concerned with the common DEGs involved in metabolism at two or three time points after parasitization, and 46 items were identified (Table S6), including the typical fatty acid synthase, serine hydrolase, glucose dehydrogenase and so on (Figure 5A). It was evidenced that 29 genes accounting for 63.04% of metabolism-related DEGs showed at least two-fold higher expressions at 2 h post-wasp parasitization. However, only 10 (21.74%) and 11 (23.91%) DEGs were respectively found to be significantly upregulated at 24 h and 48 h post-parasitization, indicating that *M. manilae* parasitization retarded the metabolism of host larvae.

The further analysis mainly focused on the immune-related genes, and only seven common DEGs were obtained from the unparasitized vs. parasitized comparisons at two or three time points (Figure 5B). Among these, the genes encoding serine protease inhibitors, scavenger receptors, hemocytins, and PPOs were identified (Table S7). The results indicated that only the serine protease inhibitor gene (LOC118262736) showed a continuously increased expression due to wasp parasitization at 2 h, 24 h, and 48 h. In contrast, albeit we recorded significant mRNA accumulations upon 2 h parasitization in the remaining six genes, there were remarkable decreases in gene expression levels after 24 h parasitization. Further analysis revealed that the differences in six gene expression levels were not significant at 48 h between the parasitized and unparasitized host larvae. Overall, these results suggested that the innate immune response of host FAW larvae was immediately activated upon *M. manilae* parasitization and was further suppressed with the development of wasp offspring.

### 3.6. Verification of the Expression Profile of DEGs

**Figure 6:**
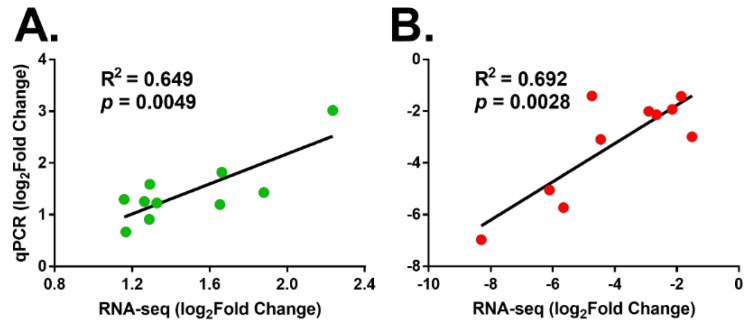
Validation of expression levels of DEGs involved in host metabolism and immunity using qPCR. (**A**) Expression levels of 10 upregulated DEGs in the parasitized FAW compared to those in unparasitized hosts using qPCR and RNA-seq data. (**B**) Expression levels of 10 downregulated DEGs in parasitized FAW compared to those in unparasitized hosts using qPCR and RNA-seq data. Log_2_ Fold change(qPCR) values were plotted against the log_2_ Fold change (FPKM) values in gene expression after wasp parasitization detected by RNA sequencing. The reference line indicates a linear relationship between the two techniques, and R denotes the Pearson correlation coefficient.

In the final part of the analysis, we validated the accuracy and reproducibility of the expression patterns of DEGs generated from the RNA-sequencing, 10 upregulated and 10 downregulated DEGs involved in host metabolism and immune were randomly selected for qPCR analysis that were expressed diversely in response to parasitization (Figure 6A,B). qPCR results indicated that the upregulated DEG expression levels showed a 1.59–8.09-fold increase post-parasitization with *M. manilae* (Figure 6A). In parallel, there were remarkable decreases in mRNA levels (0.008–0.38-fold) of several DEGs in *M. manilae* parasitized host (Figure 6B). Furthermore, the Pearson correlation analysis validated that there were significant positive correlations between the log_2_ Fold change (FPKM) values and log_2_ Fold change(qPCR) values in both upregulated (R^2^ = 0.645, *p* = 0.0049) and downregulated DEGs (R^2^ = 0.692, *p* = 0.0028).

## 4. Discussion

It has been well demonstrated that parasitoids are almost certainly the most abundant insect species, and there is at least one parasitic wasp for every non-parasitoid insect [35], especially for lepidopteran insects such as FAW. Parasitoid invasion substantially regulates the various physiological processes of host insects, mainly involving immunity, metabolism, development, and behavior, eventually killing the host insects [9,13,14,15]. Among these, metabolism regulation occupies an important position. However, few studies have been involved, and the underlying regulatory mechanisms are far from being revealed.

As a dominant larval endoparasitoid, *M. manilae* (Hymenoptera: Braconinae) is capable of parasitizing most noctuid pests, such as FAW, a major invasive pest in China [2]. Therefore, the *M. manilae*–*S. frugiperda* can serve as an ideal model for revealing the physiological interactions between parasitoids and host insects, especially in metabolism. The development of transcriptome solved many questions in host physiological responses upon parasitoid infection. Accordingly, this study was designed to determine the effects of *M. manilae* parasitization on host FAW using RNA-seq analysis. A total of 1861, 962, and 108 DEGs were respectively obtained from the parasitized groups compared with the controls at 2 h, 24 h, and 48 h. The GO functional annotations assigned most DEGs to certain metabolic pathways, including the “cellular nitrogen compound metabolic process” (GO:0034641) and “catalytic activity” (GO: 0003824). Comparisons of the findings with those of other studies, such as conducted in *Leptopilina boulardi*–*Drosophila* and *N. vitripennis*–*Sarcophaga bullata* models [22,36], confirmed that wasp parasitization altered the expression levels of host metabolism-related genes, possibly benefiting the development of offspring. Subsequent KEGG annotations also identified an abundance of DEGs associated with host metabolism, which further supported the idea of alterations in host metabolism upon parasitoid infection.

In our analysis, the DEGs annotated as lipid metabolism, and carbohydrate metabolism accounted for the vast majority. Lipid metabolism is vital to holometabolous insects for fine-tuning lipid levels during larval growth and development [37,38]. However, most parasitoid species cannot synthesize lipids as an evolutionary consequence of a parasitic lifestyle [39,40,41,42]. Alternatively, they manipulate the host insects forced to synthesize lipids to benefit themselves through environmental compensation and wasp parasitism could induce changes in host lipid metabolism. In *S. bullata–N. vitripennis* [43], *Spodoptera exigua–Microplitis pallidipes* [44], and *Cotesia vestalis–Plutella xylostella* systems [45], it was evidenced that parasitoids manipulated host systemic lipid levels to promote their development. These results corresponded well with our observations showing that DEGs involved in lipid metabolism, such as glycerolipid and sphingolipid, were enriched in KEGG pathways. Previous findings also built a strong relationship between wasp parasitism and host carbohydrate metabolism regulation. In one relevant example, the host polysaccharides were hydrolyzed upon *P. puparum* parasitism to obtain energy for the development of parasitoid offspring [15]. Similarly, it was further demonstrated that *P. vindemiae* has a vital role in regulating host glucose metabolism [46]. These cases provided a possible explanation for the enrichment of purine pentose, glutathione, and other metabolism-related DEGs in KEGG pathways. Moreover, metabolic pathways would be both upregulated to produce immune effectors, and downregulated by the wasp genes injected along with wasp eggs to counteract the upregulation of these genes.

In the category of genetic information processing, we found a large proportion of DEGs involved in RNA transport, spliceosome, and ribosome upon 2 h parasitization, reflecting the dramatic changes in RNA transcription and splicing. These results corroborated plenty of previous studies demonstrating that genetic information processing was seriously disturbed by parasitoid infections [17,18,21,22]. One unanticipated finding was that the DEGs annotated as human cancer were present in KEGG annotations. Inflammation has been recognized as a hallmark of human cancer and acts in the development and progression of most cancers [47]. The previous studies describing the inhibition of proinflammatory cytokines and the canonical NF–κB pathway essential for inflammatory responses by parasitic factors uncovered several new ideas on how wasp parasitism exhibited anti-inflammatory properties [48,49,50]. We, therefore, clarified that the host genes involved in cancer pathways were significantly enriched after *M. manilae* parasitism. Other pathways painted as cellular processes were autophagy, endocytosis, and peroxisome. This result may be explained by the fact that autophagy is a crucial pathway implicated in the conserved antimicrobial functions in insect defenses [51], sustaining their potential against pathogen and parasite invasions [52,53]. There was also clear evidence of a cross-talk between autophagy and the IMD pathway, a commonly known immune pathway triggered by wasp parasitism [54]. When it comes to the endocytosis pathway, to be active, PDV particles must gain entry into host cells, and this requires metabolic endocytosis. Another important finding was the enrichment of peroxisome-associated DEGs in KEGG pathways. It has been well established that the generation and disposal of peroxides is a critical process in insects when encountering biotic and abiotic stresses [55], such as wasp parasitization [56].

Among the common DEGs identified from the unparasitized and parasitized comparisons in at least two time points, 46 DEGs were involved in host carbohydrate metabolism, and most genes, including the representative glycosyltransferases, carboxypeptidases, and glucose 6-dehydrogenases, showed at least two-fold higher expressions at 2 h post-wasp parasitization. This study supported evidence from the previous observation, which showed that the host amylase genes were upregulated upon parasitization in *L. boulardi*–*D. melanogaster* model [57]. It was also found that 16 carbohydrate metabolism-related genes were highly expressed in the parasitized host [58], and among those, the amylases, chitinases, glucosidases, and mannosidases occupied the majority. Similarly, RNA sequencing was used to characterize the differentially expressed genes in several metabolic pathways of the host flies, and several genes responsible for glycolysis and gluconeogenesis were found in parasitized hosts [36]. Moreover, Zhou et al. (2021) screened 278 carbohydrate digestion-related genes differently expressed upon 24 h and 48 wasp parasitization based on transcriptome analysis, which was in good accordance with our present study. In a recent investigation, we reported that the glucose-6-phosphate metabolism of host *Drosophila* was inhibited after 24 h and 48 h *Pachycrepoideus vindemiae* parasitization [46], broadly supporting our present work that most metabolism-related DEGs were significantly downregulated at 24 h and 48 h post-parasitization. To further validate the expression levels of metabolism-related DEGs in parasitized FAW and to contrast with the unparasitized host, 20 genes were randomly selected for qPCR verification, which well correlated with the data of RNA-seq for quantifying the transcriptional levels of these DEGs. The altered expressions in host metabolism-related genes undoubtedly contribute to elucidating the underlying mechanism of physiological interactions between parasitic wasps and their hosts, which might be crucial for the successful development of larval wasps.

As is commonly known, the innate immune system of host insects plays a crucial role in resisting parasitoid invasion [9,59]. Of these, the immune-related genes are mainly included. In this study, we analyzed the common immune-related DEGs in at least two comparisons between the unparasitized and parasitized groups. We identified seven genes encoding a serine protease inhibitor, a scavenger receptor, two hemocytins, and three PPOs. It was encouraging to compare these findings with the previous study, which also uncovered that numerous genes encoding the components of host immune pathways and melanization cascade were differently expressed after being attacked by two parasitoids, *L. boulardi* and *L. heterotoma* [17]. Another important finding was that six immune-related genes showed increased expressions at the early stage after *M. manilae* parasitization (2 h), while the expression levels significantly decreased at 24 h post-parasitization. More specifically, serine protease inhibitors and PPOs have long been proven to regulate host melanization, thus determining the fate of host insects under the attack of parasitoids [59,60]. Gregorio et al. (2002) [61] initially reported that an immune-responsive serine protease inhibitor regulated the melanization cascade in *Drosophila* through the specific inhibition of the terminal protease. In addition, several serine protease inhibitor genes identified from wasp parasitic factors acted to suppress host melanization [62,63,64,65]. Conversely, PPO is a key enzyme synthesized as an inactive zymogen in melanin biosynthesis during melanization [59], and a strong relationship between the activation of PPOs and anti-parasitoid defensive reaction has been widely reported [9,66,67]. Additionally, scavenger receptors and hemocytins were highlighted after *M. manilae* parasitization. This result resonated with the findings that wasp parasitism perturbed the expressions of the genes implicated in the host humoral immune response [68,69,70]. We randomly selected several immune-related DEGs for qPCR verification, which showed a similar trend in expression patterns with the high throughput sequencing data in response to wasp parasitization. These results provide further support for the hypothesis that the host immune defense responses were immediately activated after the non-self-recognition of wasp eggs and were subsequently counteracted by various virulence factors introduced by immunosuppressive wasps [9], such as in *Asobara tabida*–*Drosophila* [16,18], *C. congregata–M. sexta* [20], *Pteromalus puparum–Pieris rapae* [70,71], *P. vindemiae–Drosophila* [54], *Aulacococentrum confusum–G. pyloalis* parasitic systems [21]. It’s worth mentioning that the innate immune genes induced by the act of wasp ovipositor piercing the host cuticle without injecting venom, PDV, or other factors would not have been triggered in the control group. Therefore, some DEGs in the parasitized group induced by piercing alone would likely be different from those in the control, in which the cuticle had not been pierced.

The most remarkable finding was that the expression of four genes in the host larvae continuously changed after *M. manilae* parasitization at 2 h, 24 h, and 48 h, including one unknown protein and three PPOs. Prior studies have noted the importance of PPOs, the key enzyme in the host melanin biosynthetic pathway, in encapsulating parasitoid eggs [72,73,74]. In Pteromalidae, Figitidae, and Braconidae species, such as *P. puparum*, *P. vindemiae*, *N. vitripennis*, *L. boulardi*, *C. vestalis* and *M. demolitor*, it was shown that wasp parasitization and the introduction of parasitic factors inhibited the host PO cascade in the hemolymph [54,62,64,65,75,76,77,78], which was in agreement with our present findings showing significant declines in mRNA levels of three PPO genes upon 24 h and 48 h parasitization. The downregulation of PPO implies an active mechanism from the wasp side, and it could be argued that the obtained results were possibly due to the immunosuppressive ability of *M. manilae* toward host FAW, which impairs the host immune responses and promotes successful parasitism. However, these findings may be somewhat limited by a lack of physiological verification and whether the wasp exerts immunosuppressive ability is far from demonstrated. Further studies with more focus on the underlying mechanism, which takes these variables into account, will need to be undertaken.

## 5. Conclusions

In this investigation, the aim was to assess the global gene expression of the destructive invasive pest FAW response to *M. manilae* attack at 2 h, 24 h, and 48 h based on the differentially expressed transcriptome analysis. The most obvious finding to emerge from this study was that plenty of genes were differentially expressed, among which the metabolism-related genes were mainly revealed. Also, several DEGs implicated in host immune responses were identified, showing significant mRNA accumulations at early wasp parasitization and remarkable decreases in gene expression levels at late parasitization. The insights gained from this study may be of assistance in revealing the physiological regulation of parasitoids on host insects. Specifically, this study undisputedly provides knowledge for facilitating the development of biological control practice in parasitoid–pest systems. In this scenario, two schemes can be applied. First, design novel RNAi-based insecticides targeting these DEGs to disrupt the host metabolism and immune system. A second route focuses on making genetically modified crops endogenously expressing dsRNAs of these DEGs available to dampen pest populations.

## Figures and Tables

**Table 1:** Top 10 enriched KEGG pathways in DEGs identified from the P2 vs. NP2, P24 vs. NP24, and P48 vs. NP48 groups.

KEGG Pathway		Category	Description	Number of Gene	Ratio (%)
P2 vs. NP2	ko01100	Metabolism	Metabolic pathways	119	25.32
	ko01110		Biosynthesis of secondary metabolites	37	7.87
	ko00230		Purine metabolism	30	6.38
	ko00240		Pyrimidine metabolism	23	4.89
	ko03013	Genetic Information Processing	RNA transport	27	5.74
	ko03040		Spliceosome	22	4.68
	ko03008		Ribosome biogenesis in eukaryotes	20	4.26
	ko03010		Ribosome	19	4.04
	ko05200	Human Diseases	Pathways in cancer	20	4.26
	ko04140	Cellular Processes	Autophagy-animal	17	3.62
P24 vs. NP24	ko01100	Metabolism	Metabolic pathways	50	38.17
	ko01110		Biosynthesis of secondary metabolites	20	15.27
	ko01130		Biosynthesis of antibiotics	10	7.63
	ko00561		Glycerolipid metabolism	6	4.58
	ko00564		Glycerophospholipid metabolism	6	4.58
	ko01120		Microbial metabolism in diverse environments	6	4.58
	ko00040		Pentose and glucuronate interconversions	5	3.82
	ko04146	Cellular Processes	Peroxisome	9	6.87
	ko04212	Organismal Systems	Longevity regulating pathway–worm	6	4.58
	ko05200	Human Diseases	Pathways in cancer	6	4.58
P48 vs. NP48	ko01100	Metabolism	Metabolic pathways	8	9.30
	ko01110		Biosynthesis of secondary metabolites	4	4.65
	ko00230		Purine metabolism	3	3.49
	ko00982		Drug metabolism-cytochrome P450	2	2.33
	ko00980		Metabolism of xenobiotics by cytochrome P450	2	2.33
	ko00600		Sphingolipid metabolism	2	2.33
	ko00561		Glycerolipid metabolism	2	2.33
	ko00480		Glutathione metabolism	2	2.33
	ko04144	Cellular Processes	Endocytosis	3	3.49
	ko05204	Human Diseases	Chemical carcinogenesis	2	2.33

## Data Availability

The data presented in this study are available from the corresponding author upon reasonable request.

## Supplementary Materials

The following supporting information can be downloaded at: https://www.mdpi.com/article/10.3390/insects14020100/s1, Table S1: qPCR primers used in the main text; Table S2: Overview of RNA-sequencing results; Table S3: The list of DEGs in P2 vs. NP2 comparison; Table S4: The list of DEGs in P24 vs. NP24 comparison; Table S5: The list of DEGs in P48 vs. NP48 comparison; Table S6: The common DEGs involved in metabolism in at least two time points after parasitization; Table S7: The common DEGs involved in immunity in at least two time points after parasitization.
